# Disentangling Neural Synchronization and Sustained Neural Activity in the Processing of Auditory Temporal Patterns

**DOI:** 10.3389/fnhum.2018.00497

**Published:** 2018-12-18

**Authors:** Aysha Motala, Lucila Guadalupe Caceres

**Affiliations:** ^1^School of Optometry & Vision Science Graduate Program, Cardiff University, Cardiff, United Kingdom; ^2^Sensorimotor Dynamics Lab, Department of Science and Technology, National University of Quilmes, Buenos Aires, Argentina

**Keywords:** neural synchronization, sustained neural activity, auditory rhythms, neural processing, auditory perception

Temporal regularity within sensory input, can be defined as a uniformly structured and recurring stimulation. Perceiving temporal regularity is integral to effectively perceiving the world around us, such as in speech and music perception. Indeed, natural environments constantly present our perceptual systems with different forms of temporal regularities and rhythms. Efficient sensitivity to temporal changes not only allows us to maintain a coherent perception of our experiences, but importantly, also allows us to build expectations and predict future events (Gutschalk et al., 2002; Nobre and van Ede, 2017). Previous work investigating the underlying neural mechanisms of temporal pattern perception have focused on neural synchronization (NS). This is defined as the ability of neural oscillations to synchronize with temporal regularity in external stimuli (Lakatos et al., 2008; Henry and Obleser, 2012), further suggesting that temporal regularity boosts neural activity at the same frequency as that of the external stimulus. This externally-synchronized neural activity can then be used to predict future auditory activity (Nobre and van Ede, 2017). More recently, the role of sustained activity (SA) has also been investigated in temporal regularity perception, using electroencephalography (EEG) and magnetoencephalography (Barascud et al., 2016; Southwell and Chait, 2018). For instance, detection of regularity in short auditory sequences is demarcated by increased sustained low-frequency evoked magnetoencephalographic activity, which occurs irrespective of the temporal structure (Barascud et al., 2016). The precise relationship between NS and SA is not fully understood. One suggestion is that NS allows the recognition of auditory patterns, while SA subsequently allows the processing of this information in the higher order brain regions. A recently published study by Herrmann and Johnsrude (2018) examined the relationship between NS and SA in the processing of auditory temporal patterns using EEG.

To investigate whether NS and SA co-occur, the authors used sequences of tones arranged in random and regular frequency patterns; in this case, coherent frequency modulation (FM), similar to previous experiments on SA (Barascud et al., 2016; Southwell et al., 2017). EEG recordings were performed whilst participants listened to auditory rhythms in four conditions: one contained no temporal regularity, whereas the others contained a temporal pattern. In order to study both neural processes concomitantly, two implementations were made: two of the rhythms had a sinusoidal oscillatory pattern, to observe synchronization (2.5 and 5 Hz sinusoidal FM), while high-pass filtering was omitted in studying SA, because it is a low frequency signal (Barascud et al., 2016).

Whilst the first experiment replicated previous results of increased SA when regularity occurs (Barascud et al., 2016; Southwell et al., 2017), the main finding was that both SA and NS showed increased activity, indicating the first evidence of the co-occurrence of these two processes. Herrmann and Johnsrude (2018) also showed a correlation between the effect of regularity on SA and inter-trial phase coherence, concluding that these signals are not independent. This assumption was made by the authors despite a significant correlation for only one condition, with sinusoidal oscillatory patterns for the frequency of 2.5 Hz, and no correlation for 5 Hz. Taking this difference into account, we concluded that the interdependency between the signals could be related to the frequency of the stimulus—an important distinction to understand the extent to which NS and SA can be dissociated. Further experimentation replicating this experiment using a wider range of frequencies is necessary to elucidate this relationship.

Interestingly, the finding that NS is more sensitive to regularity imposed by a coherent frequency modulation in sounds, compared to SA, indicates an indirect relationship between the two processes. This observation led the authors to investigate the extent to which the attentional state of the participant affected NS and SA in detecting temporally regular patterns. To address this, participants either attended to sounds with and without regularity (in an auditory duration categorization task) or were instructed to ignore the sounds and perform a visual multiple object tracking (MOT) task. Increased SA was observed when participants performed either a MOT task or an auditory task for sounds without temporal regularities, suggesting that attentional demands produced an increase in the response. Sohoglu and Chait (2016) have demonstrated that increased SA, due to regular patterns, is independent of attention. However, in their experiments they used a visual comparison task, while in the present MOT task the participant had to track and remember the target position. We hypothesize that the MOT task is more attentionally and/or even more cognitively demanding (Cavanagh and Alvarez, 2005). Furthermore, as the same participants made judgments in both the auditory and visual tasks in alternating blocks, the cognitive demands required to solve the MOT task could at least in part also influence the SA during the auditory task. Whether the rise in SA is due to an increase facilitated by the attentional state, or rather the recruitment of different cognitive processes for the task, [akin to observations made by Southwell et al. (2017)] remains to be clarified.

Additionally, whereas regularity-related NS activity was unaffected by visual or acoustically demanding tasks, increased SA due to regularity was only observed when participants attended to the auditory stimuli. We hypothesize that cognitive processes, such as working memory taking part during the MOT task, have an increased demand for computational resources, compared with the auditory task alone and could interfere with the sensitivity of the SA to detect regularities, compared with NS. To clarify this, we propose the use of discrimination tasks for both modalities. Interestingly, Barascud et al. (2016) demonstrated an interaction between the primary auditory cortex, the hippocampus and inferior frontal gyrus during regularity detection by SA. We suggest that the participation and activation of these areas during cognitive processes, like attention and working memory, could help to explain increased SA as well as the difference in NS.

It is noteworthy that evidence for regularity-related SA activation was only found when attention was applied during the auditory task and not during the visual task. Interpretation of this finding however, is confounded by the fact that the authors observed an important SA difference even prior to the transition to the auditory-visual comparison. Two possible interpretations for the observed differences between the auditory and visual decoy tasks could be that attending to a cognitively demanding visual task, may consequently result in the suppression of regularity-based sustained activity. Alternatively, attending to sounds could result in the enhancement of regularity-based sustained activity. Evidence that a visual task is able to impair auditory pattern detection could also suggest prioritization of visual information with respect to auditory information. Although Herrmann and Johnsrude (2018) suggest modality differences between audition and vision, this difference is only documented for SA—a process that has already been differentiated from NS. We propose an alternative hypothesis that NS and SA rely on different underlying mechanisms thereby explaining the modality of differences evidenced here.

These results provide novel evidence on NS and SA in detecting temporal regularities. Specifically, that these processes co-occur and are differentially affected by cognitive demands. The authors suggest that NS and SA reflect neural processes at different hierarchical levels, however these results alone do not inform us about the underlying neural mechanisms of these processes. It may be informative to pair EEG with neuroimaging methods, with high spatial resolution, such as functional magnetic resonance imaging, to differentiate the brain regions that mediate temporal pattern processing. Furthermore, whilst NS and SA are plausibly linked through their sensitivity to auditory regularity, evaluating the differences between SA and NS in influencing temporal pattern perception, in a behavioral sense, is also an important feat. We agree with the authors' interpretation that NS is likely to suggest more sensory-driven and automatic responses to temporal patterns, rather than SA, which is thought to be influenced by more cognitively-mediated processing. Causal perturbation of sensory cortices using Transcranial Magnetic Stimulation (TMS), or alternatively through a dual-task paradigm where one task recruits attentional mechanisms and the second employs cognitive processing (that excludes specific attentional processing), would clarify this interaction.

The findings of Herrmann and Johnsrude (2018) clarify at least in part the functional dependence between two different neural responses, NS and SA, in the processing of auditory temporal patterns. By employing coherent FM patterns and parametrically manipulating the degree of phase coherence, it was found that both NS and SA were modulated and co-occur. This novel result indicates that there are concurrent neural signals measuring temporal structure in sounds. Importantly, NS demonstrated increased sensitivity to the degree of FM phase coherence. Additionally, it was found that NS to auditory temporal regularity still occurred, despite co-occurring visual demanding distractors. However, SA was not sensitive to the same auditory regularity, which may reflect different stages of regularity processing. These results suggest that while temporal patterns induce neural activity in both responses of NS and SA, these patterns of activity are not identical and are in fact, modulated differently by cognitive demands.

## Author Contributions

AM and LGC both contributed to the conception and writing of this review. AM prepared the draft for submission.

### Conflict of Interest Statement

The authors declare that the research was conducted in the absence of any commercial or financial relationships that could be construed as a potential conflict of interest.
